# Draft whole genome sequence of *Alternaria alternata* strain P02PL2*,* an endophytic fungal species isolated from *Sclerocarya birrea*

**DOI:** 10.1128/mra.00865-24

**Published:** 2025-02-18

**Authors:** Aviwe N. Matandela, Thendo Mafuna, Sizwe I. Ndlovu

**Affiliations:** 1 Department of Biotechnology and Food Technology, Faculty of Science, University of Johannesburg 61799 , Johannesburg, Gauteng, South Africa; 2 Department of Biochemistry, Faculty of Science, Auckland Park Campus, University of Johannesburg 300829 , Auckland Park, Gauteng, South Africa; University of California Riverside, Riverside, California, USA

**Keywords:** *Alternaria alternata*, endophytes, genome analysis

## Abstract

Here, we report the draft whole genome sequence of an endophytic fungal species, *Alternaria alternata* P02PL2 isolated from *Sclerocarya birrea*. The genome of this isolate was sequenced using the MGISEQ 2000RS platform. The estimated genome size for *A. alternata* P02PL2 was 32.0 Mb with a GC content of 50.56%.

## ANNOUNCEMENT

*Alternaria alternata* are filamentous Ascomycetes belonging to Fungi Imperfecti, under the family *Dermateaceae,* and they are primarily associated with plants as plant pathogens, facultative parasites, saprophytes, or endophytes (1, 2). The availability of whole genome sequences facilitates the optimal exploitation of the bioactive compounds produced by these fungal species (3). The leaves of *Sclerocarya birrea* were collected from the South Coast region of the Durban Municipality, KwaZulu-Natal, South Africa (30° 03′ S; 30° 53′ E) as described by Fanele and Ndlovu (4). The fungal endophyte isolate was designated as P02PL2 and putatively identified based on the internal transcribed sequence (ITS) region (ITS1 and ITS4) as *A. alternata* and deposited on the GenBank under accession number OP630378 (4). The fungal slant culture was used to prepare single spore on potato dextrose agar (PDA, Neogen corporation, United Kingdom) supplemented with 50 µg/mL chloramphenicol (Sigma Aldrich, Israel) to prevent bacterial growth and cultured at 25°C for 5 days. Genomic DNA was extracted using the Norgen Plant/Fungi DNA isolation kit (25240, Norgen Biotek, Thorold, ON, Canada) following the manufacturer’s instructions. The obtained genomic DNA was sent for whole genome sequencing to the Agricultural Research Council Biotechnology platform (ARC-BTP), Onderstepoort, South Africa. The DNA libraries with an average length of 150 base pairs were prepared by mechanical shearing and size selected using magnetic beads, following the MGIEASY Universal DNA library preparation kit (MGI Tech, Co., Shenzhen, China). The library sequencing was performed using the MGISEQ-2000RS sequencing platform with 12× genome coverage. All analyses were performed using default software parameters except where otherwise stated. The quality of the raw sequence data and the removal of adapters were performed using FastQC version 0.11.9 (5). About 26,174,686 raw reads were cleaned and filtered to 26,035,308 high-quality reads for quality control processing. The low-quality reads and adapter sequences were removed using Trimmomatic version 0.39 (6). *De novo* assembly was performed using the genome assembler tool, SPAdes version 3.15.5 (7, 8). The completeness of the assembled genome was evaluated using Benchmarking Universal Single-Copy Orthologue (BUSCO) version 5.7.0 against the databases ascomycota_odb10 and sordariomycetes_odb10 (9). Repeated sequences were assessed using RepeatModeler version 2.0.5 (10), and RepeatMasker version 4.1.5 was utilized to mask the repeats. The assembled genome was annotated using the Funannotate pipeline, version 1.8.9 (11). Gene prediction was performed using *ab initio* gene prediction tool Augustus (12). Gene Ontology annotation was performed using the web tool ShinyGO version 0.77 (13). The BUSCO completeness score was 93% against 3,817 Sordariomycetes and 92% against 1,706 Ascomycetes genome data sets.

The assembled genome size was 32,005,677 bp (32.0 Mb), a GC content of 50.56%, and an N_50_ scaffold of 721,337 bp. There were 413 contigs, 9,614 RNA genes with 9,521 genes for mRNA, and 93 for tRNA. Furthermore, 6,737 protein-coding sequences were assigned for Pfam families using InterProScan 5.59–91.0. Gene ontology analysis with the top 50 terms revealed 833 biosynthetic pathways and 1,016 catalytic activities, indicating that this isolate is rich in pathways with biotechnological applications.

## Data Availability

The raw genome data is available on the National Center for Biotechnology Information (NCBI) website under BioProject accession number PRJNA1104117 and SRA number SRR28789454. This Whole Genome Shotgun project has been deposited at DDBJ/ENA/GenBank under the accession number JBDIFV000000000. The version described in this paper is version JBDIFV000000000.1 and consists of sequences JBDIFV010000001–JBDIFV010000410. The assembled genome sequence is available under the GenBank assembly accession number GCA_039700845.1. The annotation data supporting this study is available on Figshare: https://doi.org/10.6084/m9.figshare.27908586.
